# (3*R*,4*S*)-3,4-Isopropylidenedioxy-5-phenylsulfonylmethyl-3,4-dihydro-2*H*-pyrrole 1-oxide

**DOI:** 10.1107/S1600536811010737

**Published:** 2011-04-13

**Authors:** Mari Fe Flores, P. Garcia, Narciso M. Garrido, Francisca Sanz, David Diez

**Affiliations:** a Departamento de Quimica Organica, Universidad de Salamanca, Plaza de los Caidos, 37008-Salamanca. Spain; b Servicio General de Rayos X, Universidad de Salamanca, Plaza de los Caidos, 37008-Salamanca. Spain

## Abstract

The title compound, C_14_H_17_NO_5_S, was prepared by oxidation of (2*R*,3*S*,4*R*)-2-phenyl­sulfonyl­methyl-1-hy­droxy-3,4-iso­pro­pyl­idene­dioxy­pyrrolidine. Its crystal structure confirms unequivocally its configuration. Two inter­molecular C—H⋯O inter­actions help to establish the packing.

## Related literature

For the preparation, see: Flores *et al.* (2010 ▶). For the standard oxidation of hydroxyl­amines to nitro­nes with manganese dioxide, see: Cicchi *et al.* (2001 ▶). For background to organocatalysts, see: Berkessel & Groger (2005 ▶); Macmillan (2008 ▶). For analogues of the organocatalyst l-proline, see: Andrey *et al.* (2004 ▶); Cobb *et al.* (2004 ▶); Tanaka *et al.* (2004 ▶); Wang *et al.* (2005 ▶). 
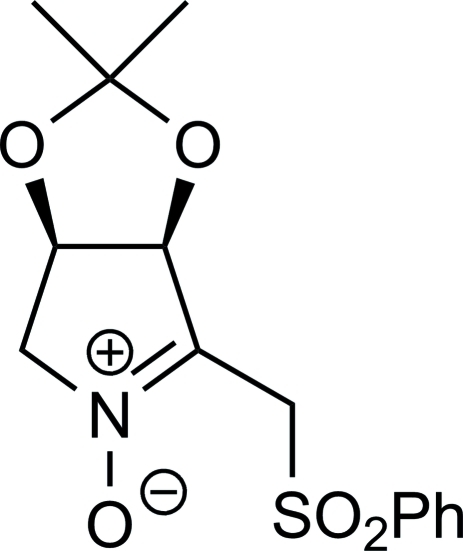


## Experimental

### 

#### Crystal data

C_14_H_17_NO_5_S
*M* *_r_* = 311.35
Orthorhombic, 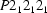

*a* = 5.6424 (2) Å
*b* = 15.5592 (7) Å
*c* = 16.9097 (8) Å
*V* = 1484.52 (11) Å^3^
*Z* = 4
Cu *K*α radiation
μ = 2.14 mm^−1^
*T* = 298 K
0.10 × 0.08 × 0.06 mm

#### Data collection

Bruker APEXII CCD area-detector diffractometer
Absorption correction: multi-scan (*SADABS*; Bruker, 2006 ▶) *T*_min_ = 0.815, *T*_max_ = 0.880
8018 measured reflections
2487 independent reflections
2159 reflections with *I* > 2σ(*I*)
*R*_int_ = 0.030

#### Refinement

*R*[*F* ^2^ > 2σ(*F* ^2^)] = 0.034
*wR*(*F* ^2^) = 0.084
*S* = 1.06
2487 reflections
192 parameters
H-atom parameters constrained
Δρ_max_ = 0.14 e Å^−3^
Δρ_min_ = −0.15 e Å^−3^
Absolute structure: Flack (1983 ▶),
Flack parameter: 0.06 (2)

### 

Data collection: *APEX2* (Bruker 2006 ▶); cell refinement: *SAINT* (Bruker 2006 ▶); data reduction: *SAINT*; program(s) used to solve structure: *SHELXS97* (Sheldrick, 2008 ▶); program(s) used to refine structure: *SHELXL97* (Sheldrick, 2008 ▶); molecular graphics: *Mercury* (Macrae *et al.*, 2008 ▶); software used to prepare material for publication: *SHELXTL* (Sheldrick, 2008 ▶).

## Supplementary Material

Crystal structure: contains datablocks global, I. DOI: 10.1107/S1600536811010737/bt5495sup1.cif

Structure factors: contains datablocks I. DOI: 10.1107/S1600536811010737/bt5495Isup2.hkl

Additional supplementary materials:  crystallographic information; 3D view; checkCIF report

## Figures and Tables

**Table 1:** Hydrogen-bond geometry (Å, °)

*D*—H⋯*A*	*D*—H	H⋯*A*	*D*⋯*A*	*D*—H⋯*A*
C4—H4⋯O4^i^	0.98	2.50	3.389 (2)	151
C12—H12⋯O3^ii^	0.93	2.51	3.270 (4)	139

Symmetry codes: (i) 
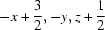
; (ii) 
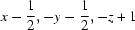
.

**Fig. 1.**
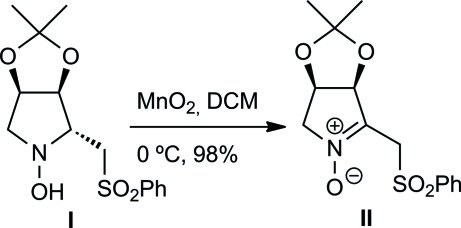
Reaction scheme.

**Fig. 2.**
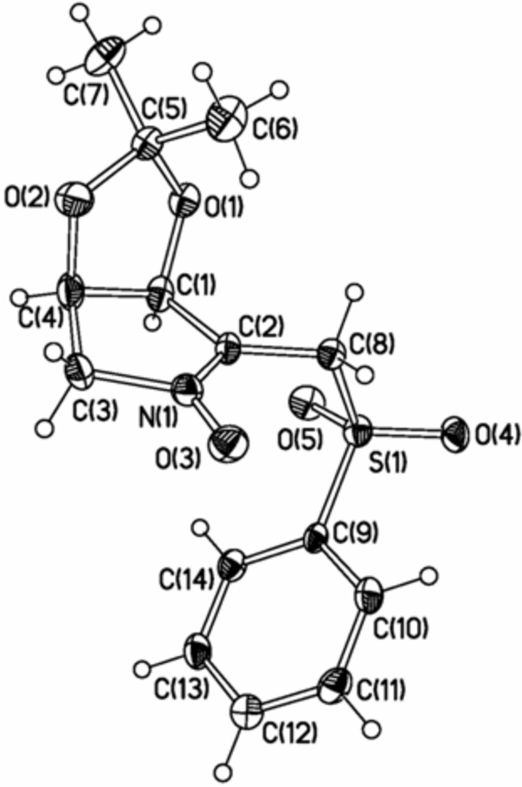
Molecular structure of C14H17NO5S. Displacement ellipsoids are drawn at the 50% probability level. Hydrogen atoms are shown as spheres of arbitrary radius.

**Fig. 3.**
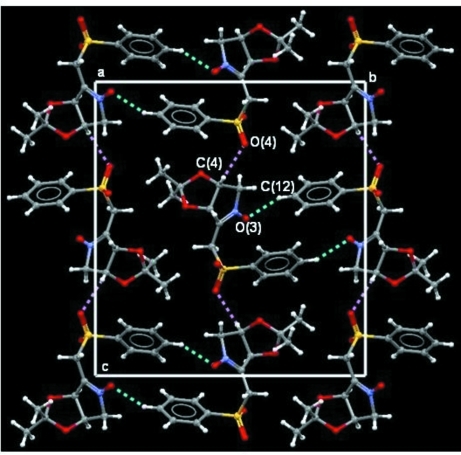
Crystal packing of C14H17NO5S view along a axis, showing intermolecular hydrogen bonding.

## supplementary crystallographic information

### Comment

Recently there has been an enormous interest in organocatalysis (Berkessel & Groger *et al.* 2005 and Macmillan, 2008). Among the many known organocatalysts *L*-proline is perhaps the one which has been most studied. This fact has led to the appearance of many analogues (Andrey *et al.*, 2004; Wang *et al.*, 2005; Cobb *et al.*, 2004; Tanaka *et al.*, 2004). In our research group we have developed new organocatalyst using nitrones as starting material (Flores *et al.*, 2010). This catalyst was obtained from chiral hydroxylamine (I) (Fig. 1). Here we communicate the oxidation of this compound into nitrone II and the crystal structure determination.

The crystal contains an unique molecule in the asymmetric unit (Fig. 2). The title molecule consists of a pyrroline-N-oxide ring with a phenylsulfonylmethylgroup and an isopropylidenedioxy group as susbtituents. All the bond lengths and angles are within the normal ranges. Statistically no diference is observed between the S1—O4 = 1.4310 (19) Å and S1—O5 = 1.4334 (18) Å distances suggesting that the two oxygen are very similar. The S1—C8 and S1—C9 bond lengths are 1.784 (2) Å and 1.755 (3) Å, respectively. The C—S—C and O—S—O angles are 104.02 (12)° and 119.46 (14)°, respectively. The large O—S—O angle and this deviation from the optimal 109.5° angle can be explained by the repulsion of the lone pairs of the oxygen placing the oxygen atoms as far away from each other as possible and thus minimizing the C—S—C angle. The molecule is twisted at the C—S bond being the C2—C8—S1—C9 torsion angle of -59.4 (7)°. The carbonyl group at atom N1 is desviated from the planar conformation with the pyrroline ring being the O3—N1—C3—C4 torsion angle of 165.9 (5)°.

Crystal packing (Fig. 3) is stabilized by two intermolecular C-H···O interactions. One   occurs between the carbon atom (C4) of the isopropylidenedioxypyrroline group and the oxygen atom (O4) of the phenylsufonylmethyl group of the neighboring molecule oriented in the opposite direction along *c* axis with d(C4-H4···O4) = 3.389 (2) Å and <C4-H4···O4> = 151°. This leads to infinite molecular chains running along the [001] direction, which are joined each other along the *b* axis by another   intermolecular interaction between the oxygen atom (O3) of the pyrroline group and the carbon atom (C12) of the phenyl group of the next molecule with d(C12-H3···O3) = 3.270 (1) Å and <C12-H3···O3> = 139°.

### Experimental

The title N-oxide, (II), was obtained by spontaneous oxidation of (2*R*,3*S*,4*R*)-2-Phenylsulfonylmethyl-1-hydroxy-3,4-isopropylidenedioxypyrrolidine (I) (Flores *et al.* 2010) in a 30% of yield. Our attention was then turned to the synthesis of this nitrone because of the important role nitrones play in organic syntheses, particularly in the field of alkaloids, nitrogen containing natural products or bioactive analogues. Taking this into account we tried the standard oxidation of hydroxylamines to nitrones with manganese dioxide according to the methodology described by Cicchi *et al.* (2001). MnO_2_ (107.2 mg, 1.11 mmol) was added to a solution of hydroxylamine I (234 mg, 0.74 mmol) in DCM (1.5 ml) at 0° C. The resulting mixture was stirred for 2 h, then it was filtered through celite; and concentrated. The resulting crude residue was purified by flash chromatography (silica gel, hexane/ EtAcO 1:1) to obtain the nitrone II (229 mg, 98%) (Flores *et al.*, 2010). Well shaped colourless single crystals were obtained by crystallization from EtOAc/Et_2_O. [α]_D_^20^ = +110.7 (c = 1.17, CHCl_3_). IR (film): 3376 (broad), 3058, 2995, 2967, 1580 cm^-1. 1^H NMR (200 MHz, CDCl_3_, δ p.p.m.): 7.94 (2*H*, m, Horto), 7.69–7.58 (3*H*, m, Hmeta Hpara), 5.58 (1*H*, d, J = 6.2 Hz, H-4), 4.87 (1H, t, J = 6.2 and 12 Hz H-3), 4.04 (2H, d, J = 4.4 Hz, H-2), 3.96 (2H, s, 2*H*-1) 1.38 (3*H*, s, Me-acetonide), 1.37 (3*H*, s, Me-acetonide). ^13^C NMR (50 MHz, CDCl_3_, δ p.p.m.): 139.7 (C—Ar), 134.62 (CH-*para*), 134.0 (C-5), 129.6 (2CH-*meta*), 128.1 (2CH-orto), 112.6 (C-acetonide), 80.8 (CH-4), 71.7 (CH-3), 68.1 (CH2–2), 52.1 (CH2–1), 27.2 (Me-acetonide), 25.8 (Me-acetonide). HRMS (EI): C_14_H_17_NO_5_S requires (*M*+Na)^+^, 334.0725, found 334.0703.

### Refinement

The hydrogen atoms were positioned geometrically  with C—H distances constrained to 0.93 Å (aromatic CH), 0.96 Å (methyl groups), 0.97 Å (methylene groups) and refined using a riding mode with *U*_iso_(H) = xUeq(C), where *x* = 1.5 for methyl H atoms and *x* = 1.2 for all other atoms.

### Crystal data

**Table:**

C_14_H_17_NO_5_S	*F*(000) = 656
*M**_r_* = 311.35	*D*_x_ = 1.393 Mg m^−^^3^
Orthorhombic, *P*2_1_2_1_2_1_	Cu *K*α radiation, λ = 1.54178 Å
Hall symbol: P 2ac 2ab	Cell parameters from 1922 reflections
*a* = 5.6424 (2) Å	θ = 3.9–55.3°
*b* = 15.5592 (7) Å	µ = 2.14 mm^−^^1^
*c* = 16.9097 (8) Å	*T* = 298 K
*V* = 1484.52 (11) Å^3^	Prismatic, colourless
*Z* = 4	0.10 × 0.08 × 0.06 mm

### Data collection

**Table:**

Bruker APEXII CCD area-detector diffractometer	2487 independent reflections
Radiation source: fine-focus sealed tube	2159 reflections with *I* > 2σ(*I*)
graphite	*R*_int_ = 0.030
phi and ω scans	θ_max_ = 67.5°, θ_min_ = 3.9°
Absorption correction: multi-scan (*SADABS*; Bruker, 2006)	*h* = −6→5
*T*_min_ = 0.815, *T*_max_ = 0.880	*k* = −18→15
8018 measured reflections	*l* = −18→19

### Refinement

**Table:**

Refinement on *F*^2^	Secondary atom site location: difference Fourier map
Least-squares matrix: full	Hydrogen site location: inferred from neighbouring sites
*R*[*F*^2^ > 2σ(*F*^2^)] = 0.034	H-atom parameters constrained
*wR*(*F*^2^) = 0.084	*w* = 1/[σ^2^(*F*_o_^2^) + (0.0335*P*)^2^ + 0.1514*P*] where *P* = (*F*_o_^2^ + 2*F*_c_^2^)/3
*S* = 1.06	(Δ/σ)_max_ < 0.001
2487 reflections	Δρ_max_ = 0.14 e Å^−^^3^
192 parameters	Δρ_min_ = −0.15 e Å^−^^3^
0 restraints	Absolute structure: Flack (1983), 907 Friedel pairs
Primary atom site location: structure-invariant direct methods	Flack parameter: 0.06 (2)

### Special details

**Table:**

Geometry. All e.s.d.'s (except the e.s.d. in the dihedral angle between two l.s. planes) are estimated using the full covariance matrix. The cell e.s.d.'s are taken into account individually in the estimation of e.s.d.'s in distances, angles and torsion angles; correlations between e.s.d.'s in cell parameters are only used when they are defined by crystal symmetry. An approximate (isotropic) treatment of cell e.s.d.'s is used for estimating e.s.d.'s involving l.s. planes.
Refinement. Refinement of *F*^2^ against ALL reflections. The weighted *R*-factor *wR* and goodness of fit *S* are based on *F*^2^, conventional *R*-factors *R* are based on *F*, with *F* set to zero for negative *F*^2^. The threshold expression of *F*^2^ > σ(*F*^2^) is used only for calculating *R*-factors(gt) *etc*. and is not relevant to the choice of reflections for refinement. *R*-factors based on *F*^2^ are statistically about twice as large as those based on *F*, and *R*- factors based on ALL data will be even larger.

### Fractional atomic coordinates and isotropic or equivalent isotropic displacement parameters (Å^2^)

**Table:**

	*x*	*y*	*z*	*U*_iso_*/*U*_eq_	
S1	0.89076 (11)	0.02630 (4)	0.36234 (4)	0.05643 (18)	
O1	0.8060 (3)	0.16596 (13)	0.57625 (11)	0.0614 (5)	
O2	0.9722 (5)	0.10911 (14)	0.68680 (13)	0.0894 (7)	
O3	1.3072 (4)	−0.05736 (13)	0.53437 (13)	0.0813 (6)	
O4	0.9927 (4)	0.05353 (13)	0.28901 (11)	0.0739 (6)	
O5	0.6496 (3)	0.04808 (13)	0.37996 (14)	0.0738 (6)	
N1	1.1197 (4)	−0.01709 (14)	0.55737 (13)	0.0619 (6)	
C1	0.8010 (4)	0.07555 (18)	0.56728 (16)	0.0587 (7)	
H1	0.6470	0.0557	0.5477	0.070*	
C2	0.9998 (4)	0.04067 (16)	0.51895 (15)	0.0521 (6)	
C3	1.0217 (6)	−0.03387 (19)	0.63738 (19)	0.0805 (8)	
H3A	1.1460	−0.0341	0.6770	0.097*	
H3B	0.9384	−0.0884	0.6390	0.097*	
C4	0.8538 (5)	0.0402 (2)	0.64986 (17)	0.0729 (8)	
H4	0.7099	0.0231	0.6782	0.088*	
C5	0.9533 (4)	0.18574 (16)	0.64286 (17)	0.0549 (6)	
C6	1.1963 (5)	0.2119 (3)	0.6143 (2)	0.0922 (11)	
H6A	1.2918	0.2289	0.6586	0.138*	
H6B	1.1819	0.2592	0.5782	0.138*	
H6C	1.2698	0.1642	0.5878	0.138*	
C7	0.8367 (6)	0.2541 (2)	0.6910 (2)	0.0864 (10)	
H7A	0.6857	0.2338	0.7094	0.130*	
H7B	0.8143	0.3045	0.6591	0.130*	
H7C	0.9351	0.2680	0.7355	0.130*	
C8	1.0714 (4)	0.06909 (17)	0.43949 (14)	0.0520 (6)	
H8A	1.2345	0.0520	0.4304	0.062*	
H8B	1.0648	0.1313	0.4374	0.062*	
C9	0.9226 (4)	−0.08537 (16)	0.37208 (15)	0.0523 (6)	
C10	1.1198 (5)	−0.12509 (19)	0.33956 (16)	0.0653 (7)	
H10	1.2338	−0.0933	0.3127	0.078*	
C11	1.1429 (5)	−0.2125 (2)	0.3480 (2)	0.0754 (9)	
H11	1.2732	−0.2400	0.3257	0.090*	
C12	0.9786 (6)	−0.26022 (19)	0.38851 (18)	0.0705 (8)	
H12	0.9981	−0.3193	0.3941	0.085*	
C13	0.7836 (5)	−0.21963 (19)	0.42093 (19)	0.0703 (8)	
H13	0.6707	−0.2516	0.4481	0.084*	
C14	0.7551 (4)	−0.13237 (18)	0.41327 (17)	0.0609 (7)	
H14	0.6245	−0.1051	0.4356	0.073*	

### Atomic displacement parameters (Å^2^)

**Table:**

	*U*^11^	*U*^22^	*U*^33^	*U*^12^	*U*^13^	*U*^23^
S1	0.0530 (3)	0.0692 (4)	0.0471 (3)	−0.0058 (3)	−0.0062 (3)	0.0034 (3)
O1	0.0476 (9)	0.0863 (13)	0.0503 (11)	0.0057 (8)	−0.0094 (9)	0.0002 (9)
O2	0.1365 (19)	0.0753 (12)	0.0565 (12)	−0.0047 (13)	−0.0356 (13)	0.0064 (10)
O3	0.0906 (14)	0.0759 (13)	0.0774 (15)	0.0194 (11)	0.0000 (12)	0.0008 (11)
O4	0.0948 (13)	0.0849 (13)	0.0421 (11)	−0.0213 (11)	−0.0065 (11)	0.0110 (9)
O5	0.0455 (9)	0.0819 (13)	0.0939 (16)	0.0066 (8)	−0.0109 (10)	−0.0017 (11)
N1	0.0731 (13)	0.0599 (12)	0.0528 (13)	−0.0067 (12)	0.0011 (13)	0.0040 (11)
C1	0.0440 (13)	0.0819 (18)	0.0503 (16)	−0.0131 (12)	0.0022 (12)	−0.0013 (14)
C2	0.0494 (12)	0.0638 (15)	0.0431 (13)	−0.0127 (12)	−0.0020 (12)	0.0028 (12)
C3	0.116 (2)	0.0678 (17)	0.0573 (17)	−0.0182 (17)	0.0092 (19)	0.0161 (16)
C4	0.0835 (18)	0.087 (2)	0.0485 (15)	−0.0228 (16)	0.0111 (16)	0.0062 (15)
C5	0.0470 (12)	0.0704 (15)	0.0471 (14)	0.0057 (11)	−0.0093 (13)	0.0006 (13)
C6	0.0543 (16)	0.135 (3)	0.087 (3)	−0.0178 (17)	−0.0027 (17)	−0.017 (2)
C7	0.077 (2)	0.117 (3)	0.065 (2)	0.0361 (18)	−0.0159 (17)	−0.0214 (19)
C8	0.0454 (13)	0.0655 (14)	0.0451 (14)	−0.0080 (11)	0.0019 (12)	0.0038 (11)
C9	0.0469 (13)	0.0682 (14)	0.0418 (14)	−0.0061 (11)	0.0018 (12)	−0.0013 (11)
C10	0.0524 (14)	0.0823 (18)	0.0612 (17)	−0.0056 (14)	0.0119 (15)	0.0050 (14)
C11	0.0662 (17)	0.0833 (19)	0.077 (2)	0.0129 (15)	0.0121 (18)	−0.0042 (16)
C12	0.0748 (18)	0.0687 (16)	0.068 (2)	−0.0018 (14)	−0.0025 (17)	0.0036 (15)
C13	0.0686 (17)	0.080 (2)	0.062 (2)	−0.0203 (15)	0.0052 (17)	0.0047 (15)
C14	0.0471 (13)	0.0774 (18)	0.0581 (18)	−0.0093 (13)	0.0092 (14)	−0.0027 (14)

### Geometric parameters (Å, °)

**Table:**

S1—O4	1.4310 (19)	C5—C6	1.510 (4)
S1—O5	1.4334 (18)	C6—H6A	0.9600
S1—C9	1.755 (3)	C6—H6B	0.9600
S1—C8	1.784 (2)	C6—H6C	0.9600
O1—C1	1.415 (3)	C7—H7A	0.9600
O1—C5	1.433 (3)	C7—H7B	0.9600
O2—C5	1.409 (3)	C7—H7C	0.9600
O2—C4	1.409 (4)	C8—H8A	0.9700
O3—N1	1.290 (3)	C8—H8B	0.9700
N1—C2	1.299 (3)	C9—C14	1.383 (3)
N1—C3	1.485 (4)	C9—C10	1.387 (3)
C1—C2	1.490 (4)	C10—C11	1.374 (4)
C1—C4	1.530 (4)	C10—H10	0.9300
C1—H1	0.9800	C11—C12	1.371 (4)
C2—C8	1.471 (3)	C11—H11	0.9300
C3—C4	1.506 (5)	C12—C13	1.382 (4)
C3—H3A	0.9700	C12—H12	0.9300
C3—H3B	0.9700	C13—C14	1.373 (4)
C4—H4	0.9800	C13—H13	0.9300
C5—C7	1.492 (4)	C14—H14	0.9300
			
O4—S1—O5	119.46 (14)	C7—C5—C6	112.5 (3)
O4—S1—C9	109.47 (13)	C5—C6—H6A	109.5
O5—S1—C9	108.16 (12)	C5—C6—H6B	109.5
O4—S1—C8	107.06 (11)	H6A—C6—H6B	109.5
O5—S1—C8	107.58 (13)	C5—C6—H6C	109.5
C9—S1—C8	104.02 (12)	H6A—C6—H6C	109.5
C1—O1—C5	108.01 (19)	H6B—C6—H6C	109.5
C5—O2—C4	112.0 (2)	C5—C7—H7A	109.5
O3—N1—C2	127.8 (2)	C5—C7—H7B	109.5
O3—N1—C3	119.7 (2)	H7A—C7—H7B	109.5
C2—N1—C3	112.5 (2)	C5—C7—H7C	109.5
O1—C1—C2	113.9 (2)	H7A—C7—H7C	109.5
O1—C1—C4	104.8 (2)	H7B—C7—H7C	109.5
C2—C1—C4	102.9 (2)	C2—C8—S1	113.50 (17)
O1—C1—H1	111.6	C2—C8—H8A	108.9
C2—C1—H1	111.6	S1—C8—H8A	108.9
C4—C1—H1	111.6	C2—C8—H8B	108.9
N1—C2—C8	121.4 (2)	S1—C8—H8B	108.9
N1—C2—C1	111.7 (2)	H8A—C8—H8B	107.7
C8—C2—C1	126.8 (2)	C14—C9—C10	120.8 (3)
N1—C3—C4	103.1 (2)	C14—C9—S1	120.1 (2)
N1—C3—H3A	111.1	C10—C9—S1	119.10 (19)
C4—C3—H3A	111.1	C11—C10—C9	118.4 (3)
N1—C3—H3B	111.1	C11—C10—H10	120.8
C4—C3—H3B	111.1	C9—C10—H10	120.8
H3A—C3—H3B	109.1	C12—C11—C10	121.6 (3)
O2—C4—C3	110.3 (3)	C12—C11—H11	119.2
O2—C4—C1	102.9 (2)	C10—C11—H11	119.2
C3—C4—C1	105.6 (2)	C11—C12—C13	119.3 (3)
O2—C4—H4	112.5	C11—C12—H12	120.4
C3—C4—H4	112.5	C13—C12—H12	120.4
C1—C4—H4	112.5	C14—C13—C12	120.5 (3)
O2—C5—O1	106.07 (19)	C14—C13—H13	119.8
O2—C5—C7	110.4 (3)	C12—C13—H13	119.8
O1—C5—C7	109.1 (2)	C13—C14—C9	119.4 (3)
O2—C5—C6	109.1 (2)	C13—C14—H14	120.3
O1—C5—C6	109.4 (2)	C9—C14—H14	120.3

### Hydrogen-bond geometry (Å, °)

**Table:**

*D*—H···*A*	*D*—H	H···*A*	*D*···*A*	*D*—H···*A*
C4—H4···O4^i^	0.98	2.50	3.389 (2)	151
C12—H12···O3^ii^	0.93	2.51	3.270 (4)	139

Symmetry codes: (i) −*x*+3/2, −*y*, *z*+1/2; (ii) *x*−1/2, −*y*−1/2, −*z*+1.
